# Paradoxical Reaction to Antituberculosis Therapy Mimicking Tumor Progression in Lung Cancer Patient

**DOI:** 10.3390/diagnostics15040472

**Published:** 2025-02-14

**Authors:** Eunkyoung Choi, Yong-An Chung, Ju Sang Kim, Jinkyoung Oh

**Affiliations:** 1 Department of Radiology, Incheon St. Mary’s Hospital, College of Medicine, The Catholic University of Korea, Seoul 06591, Republic of Korea; eet0224@gmail.com (E.C.); nucmedkr@gmail.com (Y.-A.C.); 2 Division of Pulmonary and Critical Care Medicine, Department of Internal Medicine, Incheon St. Mary’s Hospital, College of Medicine, The Catholic University of Korea, Seoul 06951, Republic of Korea; kimjusang@catholic.ac.kr

**Keywords:** tuberculosis, paradoxical reaction, antitubercular agents, lung neoplasms, neoplasm metastasis, fluorodeoxyglucose F18, radionuclide imaging

## Abstract

We describe the case of a 67-year-old man with lung cancer, who developed pulmonary tuberculosis (TB) following chemotherapy and subsequently exhibited a paradoxical reaction on positron emission tomography/computed tomography (PET/CT) after initiating antituberculosis therapy. While pulmonary consolidations improved with antituberculosis treatment, newly detected hypermetabolic mediastinal lymph nodes appeared on PET/CT. Based on the clinical course, we provisionally concluded that the mediastinal lymphadenopathy represented a paradoxical reaction. Endobronchial ultrasound-guided transbronchial needle aspiration (EBUS-TBNA) confirmed the diagnosis of TB. Clinicians added steroids and continued the antituberculosis medication, and follow-up PET/CT showed complete resolution of these lesions. This case highlights the importance of recognizing paradoxical reactions to antituberculosis therapy, when restaging PET/CT reveals divergent findings, with some tumor foci responding and other lesions appearing to be progressing.

**Figure 1:**
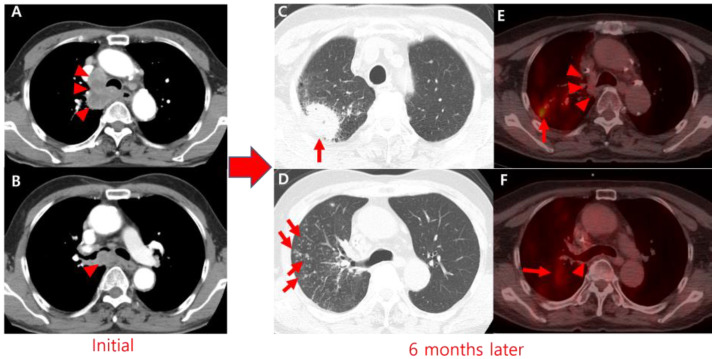
(**A**,**B**) A 67-year-old man with small-cell lung cancer (arrowheads) who underwent chemotherapy with etoposide and cisplatin. (**C**,**D**) After 6 months, he developed a cough and sputum production. Chest computed tomography (CT) (120 kVp; automatic exposure control; slice thickness, 3 mm) and 2-deoxy-2-[^18^F] fluoro-D-glucose (FDG) positron emission tomography/computed tomography (PET/CT) (Discovery STe, General Electric Healthcare, Milwaukee, WI, USA; 5.5 MBq/kg of FDG; uptake time, 60 min; iterative reconstruction with CT-based attenuation correction) were performed. Chest CT revealed multifocal consolidations and centrilobular nodules in the right upper lobe (RUL, red arrows). Pulmonary tuberculosis (TB) was confirmed by positive sputum cultures for *Mycobacterium tuberculosis*, and antituberculosis therapy was initiated. (**E**,**F**) PET/CT demonstrated increased FDG uptake (red arrows) in the newly developed consolidations and nodules, while the small-cell lung cancer had resolved (arrowheads).

**Figure 2:**
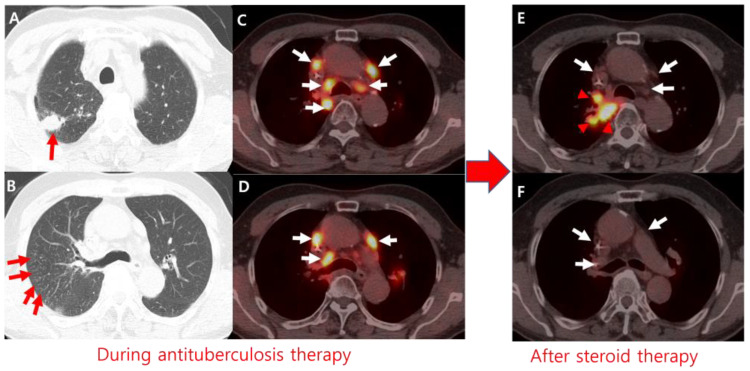
(**A**,**B**) After 3 months of antituberculosis therapy, chest computed tomography (CT) showed improvement in pulmonary tuberculosis (TB) lesions in both lungs (red arrows). (**C**,**D**) Positron emission tomography/computed tomography (PET/CT) revealed newly developed hypermetabolic, enlarged lymph nodes (white arrows) in the mediastinum, hilar, and peribronchial regions. Bronchoscopy showed no endobronchial lesions, and both transbronchial lung biopsy specimens and bronchoalveolar lavage fluid tested negative for acid-fast bacilli (AFB) on smear and culture. Based on the clinical course, two possibilities were considered: lung cancer progression or a paradoxical reaction to antituberculosis therapy. To rule out tumor progression, EBUS-TBNA was performed, confirming a diagnosis of TB lymphadenitis. (**E**,**F**) The patient was started on prednisolone (10 mg daily) alongside continued antituberculosis therapy. Follow-up 2-[fluorine-18]fluoro-2-deoxy-d-glucose (FDG) PET/CT showed regression of the hypermetabolic lymphadenopathy (white arrows). TB lymphadenitis on endobronchial ultrasound-guided transbronchial needle aspiration (EBUS-TBNA), along with the resolution of lymphadenopathy following steroid administration, supported the diagnosis of a paradoxical reaction rather than lung cancer progression. However, a newly developed hypermetabolic mass in the right upper lobe (arrowheads) was diagnosed as recurrent small-cell lung cancer via transbronchial lung biopsy. The patient underwent chemotherapy for tumor recurrence. Despite the treatment, the patient progressed and expired one year later. The paradoxical reaction to antituberculosis therapy refers to the clinical or radiological worsening of pre-existing TB lesions or the emergence of new lesions in patients who initially show improvement on antituberculosis medication. This reaction is not attributable to TB progression [1,2,3]. Although its exact mechanism remains unclear, it is hypothesized to result from a cell-mediated, disproportionate, and dysregulated inflammatory response triggered by immune system recovery. The suppressed immunity, once no longer exposed to the antigens of the tubercle bacilli, recovers and activates a host reaction [4,5]. Paradoxical reactions are frequently reported in patients receiving highly active antiretroviral therapy for HIV infection [6,7,8]. These reactions, known as paradoxical TB-immune reconstitution inflammatory syndrome (TB-IRIS), occur when clinical deterioration cannot be explained by other factors. TB-IRIS is driven by a dysregulated immune response to TB antigens during immune reconstitution following antiretroviral therapy initiation. It involves a synergistic interaction between innate and adaptive immune mechanisms, characterized by uncontrolled CD4 T-cell expansion, dysregulated cytokine production, and impaired macrophage function. This leads to an exaggerated inflammatory response, even against dead or dying bacilli, resulting in severe inflammation [4]. The interpretation of PET/CT findings can be challenging in patients with coexisting conditions, such as synchronous malignancies, infectious diseases, or treatment-related inflammation [9]. To the best of our knowledge, this is the first reported case of a paradoxical reaction identified on FDG PET/CT following the initiation of antituberculosis therapy. Previous reports have primarily described paradoxical reactions based on radiological findings observed on CT scans [2,8]. As demonstrated in our case, paradoxical reactions should be considered when restaging PET/CT shows divergent findings, with some tumor foci responding and others seen progressing.

## Data Availability

No new data were created or analyzed in this study.
